# Fostering Conversations at Your Academic Poster: Creating Space for Comments and Interaction

**DOI:** 10.1212/NE9.0000000000200211

**Published:** 2025-03-07

**Authors:** David Eli Freedman, Jiwon Oh, Anthony Feinstein

**Affiliations:** 1 Department of Psychiatry, Temerty Faculty of Medicine, University of Toronto, Ontario, Canada;; 2 Department of Psychiatry, Sunnybrook Health Sciences Centre, Toronto, Ontario, Canada;; 3 Division of Neurology, Department of Medicine, St. Michael's Hospital, Toronto, Ontario, Canada; and; 4 Division of Neurology, Temerty Faculty of Medicine, University of Toronto, Ontario, Canada.

Preparing our poster for the European Committee for Treatment and Research in Multiple Sclerosis (ECTRIMS) 2024 Annual Meeting, we asked a simple question, “How can we foster conversations at our poster presentation?” As most readers know, poster presentations are a traditional method of communicating research at academic conferences. Yet, few conference delegates attend poster presentations,^1,2^ and of those who do, content recall is exceptionally poor.^1^ Attendees may grasp the research but leave presentations without engaging with the presenter or understanding the study's implications. Prompted by these gaps, we tried a new poster style at ECTRIMS 2024: inviting attendees to write their interpretations on our poster.

**Figure 1:**
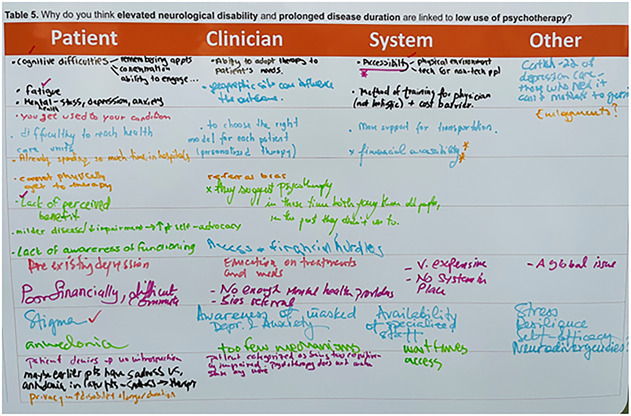
Interactive Section of the Poster Conference delegates at the European Committee for Treatment and Research in Multiple Sclerosis 2024 Annual Meeting filled out the poster table (using provided markers) with potential explanations of the findings. Subheadings of potential response categories were provided to help attendees organize their thoughts.

Our poster finding was that in people with multiple sclerosis, elevated disability and prolonged disease duration were associated with reduced utilization of psychotherapy.^3,4^ Mostly, the poster followed a standard structure: title, introduction, methods, results, conclusions, and references. However, sandwiched between results and conclusions, we included a blank table. In this table, as displayed in Figure 1, we asked attendees, “Why do you think elevated neurological disability and prolonged disease duration are linked to low use of psychotherapy?” We dedicated approximately 40% of the poster space for responses (outlined in eFigure 1). Many attendees paused to consider their answers or to ask questions. When ready, they picked up markers and wrote ideas. Several attendees even took photographs, indicating that they had never seen a similar poster.

This poster style had several benefits. Perhaps owing to its novelty, many people approached the poster, disseminating our findings. Attendees reflected on whether the results were driven by patient variables (e.g., associated cognitive difficulties or changes in self-efficacy or interest), clinician factors (e.g., referral bias), or system barriers (e.g., environmental or financial accessibility). Some questioned whether disability severity influences the response to traditional psychotherapy. While it is beyond this article's scope to discuss the supporting evidence (or lack thereof) for these ideas, it highlights how this poster style can capitalize on a large academic conference to generate an array of ideas.

Potential facilitators of the positive reception for this poster included its unconventionality, its clear concept, and the opportunities for discussion.^2,5^ Dale and Kline^6^ found that interactive conference posters on bioenergy sustainability similarly attracted attention and bolstered productive exchanges. Although others have used technology to enhance interaction at poster presentations, we have not observed chances to write on posters at other medical conferences.

There are caveats to consider. The simple methodology allowed us to condense our study description to approximately 300 words (excluding the title and references) and 4 small tables to leave room for responses. This may not be feasible for some specialized topics. In addition, this approach to surveying conference delegates does not replace rigorous research. Nevertheless, this poster design increased engagement. It produced novel ideas about psychotherapy access, which may improve local care and shape future studies. This format is just one alternative to the classical poster presentation. Still, we hope that this article sparks imaginative ideas in readers about how to expand the traditional approaches to research communication at neurology conferences.
